# Safety and Efficacy of Moderate-Intensity Stereotactic Body Radiation Therapy for Ultra-Central Lung Tumor

**DOI:** 10.3390/medicina60040538

**Published:** 2024-03-26

**Authors:** Chai Hong Rim, Won Sup Yoon, Sunmin Park

**Affiliations:** Department of Radiation Oncology, Korea University Ansan Hospital, Ansan-si 15355, Gyeonggi-do, Republic of Korea; cusion3@naver.com (C.H.R.); irionyws@korea.ac.kr (W.S.Y.)

**Keywords:** stereotactic body radiotherapy, ultra-central tumor, feasibility, radiotherapy

## Abstract

*Background and Objectives:* Ultra-central (UC) lung tumors are defined as those abutting the proximal bronchial tree. Stereotactic body radiation therapy (SBRT) for UC tumors is difficult because of concerns about severe toxicities. Therefore, we report the safety and efficacy of moderate-intensity SBRT for UC tumors at our institution. *Materials and Methods:* From January 2017 to May 2021, we treated 20 patients with UC tumors with SBRT at a dose of 45–60 Gy in 10 fractions. The primary endpoints were local control (LC) and overall survival (OS). *Results***:** The median follow-up time was 15.8 months (range: 2.7–53.8 months). Ten of the 20 patients (50.0%) showed a complete response, five (25.0%) had a partial response, two (10.0%) had stable disease, and three (15.0%) showed progressive disease (PD). The response and disease control rates were 75.0% and 85.0%, respectively. Patients with PD showed local progression at median 8.3 months (range: 6.8–19.1 months) after SBRT. One-year and 2-year OS rates were 79.4% and 62.4%, respectively. One-year and 2-year LC rates are 87.1% and 76.2%, respectively. Eight patients died due to a non-radiation therapy related cause. One patient experienced grade 5 massive hemoptysis 6 months after SBRT, resulting in death. One patient experienced grade 2 esophageal pain and two experienced grade 2 radiation pneumonitis. Otherwise, no grade 3 or higher toxicities were reported. *Conclusions:* Moderate-intensity SBRT offers effective control of UC tumors and is a well-tolerated treatment for such tumors.

## 1. Introduction

Stereotactic body radiotherapy (SBRT) is a radiotherapy modality that precisely focuses and irradiates high-dose radiation on tumors in a short period of time [1]. It is a non-invasive method for the radical treatment of localized lung cancer [2]. SBRT is commonly performed in patients who are difficult to operate on owing to comorbidities, providing similar results to surgical resection in terms of local control (LC) [3]. SBRT has high feasibility when the tumor is located in the peripheral lung parenchyma. However, when performing SBRT on a centrally located tumor, concerns exist regarding complications due to bystander irradiation on adjacent major organs [4]. In particular, tumors adjacent to the proximal bronchial tree (PBT) have been contraindicated due to the possibility of bronchial bleeding after SBRT [5]. However, several researchers have obtained favorable results by using the protracted SBRT regimen, which is biologically less toxic, and identifying and avoiding high-risk groups of such complications (e.g., anticoagulants, bevacizumab, and large tumors) [6]. Since ultra-central (UC) tumors adjacent to the PBT may require a burdensome operation, such as pneumonectomy, non-invasive curative attempts through SBRT have significant therapeutic benefits [7,8].

Moderate-intensity SBRT can provide therapeutic benefits by reducing toxicities through enhanced normal tissue repair mechanisms, in contrast to conventional SBRT, which is usually irradiated within 3–4 times [9]. At our institution, moderate-intensity SBRT was prescribed, with 45–60 Gy divided into 10 fractions, when patients with UC lung tumors had difficulty undergoing or refused surgery. In this study, we report the effectiveness and feasibility of moderate-intensity SBRT in prospectively recruited patients with UC lung tumors.

## 2. Materials and Methods

### 2.1. Study Cohort

An Institutional Review Board-approved chart review was performed. The Institutional Review Board of Korea University Medical Center Ansan Hospital approved the study for patients with UC tumors who were treated with SBRT between January 2017 and May 2021 (IRB number: 2018AS0175). The Institutional Review Board waived the requirement for informed consent because the analysis used anonymous clinical data that were obtained after each patient had agreed to treatment. The study is registered in the Clinical Research information Service (CRIS) of South Korea (registration No.: KCT0006736), which is part of the World Health Organization (WHO) International Clinical Trials Registry Platform. Patients with UC tumors who underwent moderate intensity SBRT, defined as 45–60 Gy of targeted radiotherapy in 10 fractions, were prospectively recruited. The PBT was defined as a 2 cm radius around the main trachea–bronchial tree, as defined by the Radiation Therapy Oncology Group (RTOG) series (RTOG 0236, 0618, and 0813) [10,11,12]. UC lung tumor was defined as a tumor abutting or invading the PBT. We previously reported the preliminary results of oncologic outcomes [8], and, in this study, we update and evaluate the safety and efficacy of moderate-intensity SBRT for UC tumors at our institution.

### 2.2. Evaluation and Statistics

Primary endpoints of this study were LC and overall survival (OS). The secondary endpoint was a complication of grade 2 or higher. Tumor response and adverse events were assessed according to the Response Evaluation Criteria in Solid Tumors version 1.1 [13] and Common Terminology Criteria for Adverse Events (CTCAE; version 4.03). Acute toxicity (<90 days after treatment) was evaluated weekly during the treatment by the radiation oncologist at the outpatient clinic. Late toxicity (>90 days after treatment) was scored according to the CTCAEv4.03. After treatment, all patients had regular clinical and imaging follow-ups at 3- or 6-month intervals. A detailed questionnaire on clinical status, as well as a physical examination, was administered during each follow-up, along with chest computed tomography (CT). This study was conducted according to the trial protocol. Follow-up time was calculated from the end of radiotherapy. The probability of cumulative survival was estimated using the Kaplan–Meier method. All statistical analyses were performed using IBM SPSS Statistics for Windows, version 21.0 (IBM Corp., Armonk, NY, USA). We considered *p* values < 0.05 as statistically significant.

### 2.3. SBRT Procedure

The simulation and target volume delineation were identical to those used in our previous study [8]. Briefly, four-dimensional CT (4DCT) was acquired in the axial cine mode with a slice thickness of 2–3 mm using a Philips Brilliance Big Bore CT (Philips Healthcare, Cleveland, OH, USA), with patients in the supine position and their arms above their heads. The 4DCT data were sorted into 10 phases of CT images, with 0% and 50% representing the maximum inhalation and exhalation of the breathing cycle, respectively. The Body Pro-Lok™ (Body Pro-Lok, CIVCO, Kalona, IA, USA) system was used, with the compression belt positioned at the mid-abdomen. The patients were immobilized by Vac-Lok (Kikwang Medical, Hanam-si, Gyeonggi-do, Korea) and wing board (CIVCO, USA). To analyze the patients’ respiratory data, a real-time position management system was used. All CT scan data were transferred to an Eclipse V.8.9 Treatment Planning System (Varian Medical Systems, Palo Alto, CA, USA). A total dose of 45–60 Gy was prescribed in 10 fractions, with the D_max_ (the maximum point of dose in the target volume) in the target volume not exceeding 110% of the prescribed dose. The setup for treatment was verified by a physician; the bony structures and gross tumor volume were matched by an onboard con-beam CT image.

## 3. Results

### 3.1. Patient Characteristics

Between January 2017 and May 2021, 21 patients were treated with SBRT for UC tumors. Among them, one patient received 50 Gy in 10 fractions for a 2.5 cm tumor obstructing the left upper lobe (LUL) main bronchus. The tumor completely regressed at 7 months, and the patient refused further follow-up. However, the tumor relapsed during follow-up at another hospital where the patient received an additional SBRT with an unknown dose. The patient revisited our hospital at 27 months due to hemoptysis but expired during hospitalization. Consequently, this patient was excluded from this study due to protocol violation. Therefore, 20 patients were included in the present study. All patients received 45–60 Gy of SBRT in 10 fractions, with a median dose of 55 Gy in 10 fractions. Only one patient received 45 Gy and the others received 50–60 Gy in 10 fractions. The longest diameter of the tumors was 3.6–9.2 cm (median: 3.5 cm) and the volume was 3.6–87.4 cm^3^ (median: 22.2 cm^3^). The median planned D_max_ was 106.7% (range, 102.1–108.5%). Six patients (30%) received SBRT as a definitive aim and 13 (65%) received SBRT as a salvage treatment for recurrent disease. Two patients who had undergone salvage SBRT had a history of definitive concurrent chemoradiotherapy (patient no. 7 and 11). One patient received SBRT for the consolidation of small cell lung cancer (SCLC) after chemotherapy.

Most patients were male (87.5%), with a median age of 65 years (range, 48–77 years) at treatment. Eighteen patients (90%) had non-small cell lung cancer (NSCLC), of which eight had squamous cell cancer (SqCC), eight had adenocarcinoma, and two had large cell neuroendocrine carcinoma. Additionally, one patient had SCLC and one was radiologically diagnosed with lung cancer, which was not pathologically confirmed. Regarding underlying lung disease, five patients had chronic obstructive lung disease and one had interstitial lung disease. Two patients had a history of old tuberculosis without deterioration of lung function. The characteristics of patients are summarized in Table 1.

### 3.2. Local Control, OS, and Toxicities

**Figure 1:**
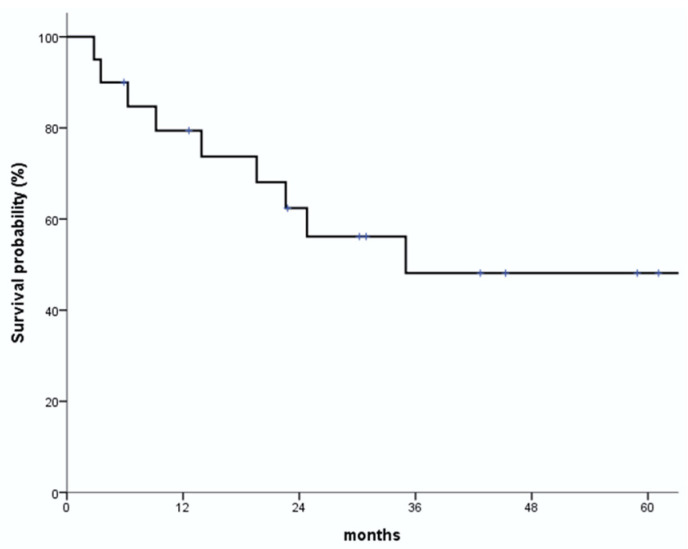
Overall survival (OS) rates. One-year and 2-year OS rates are 79.4% and 62.4%, respectively.

**Figure 2:**
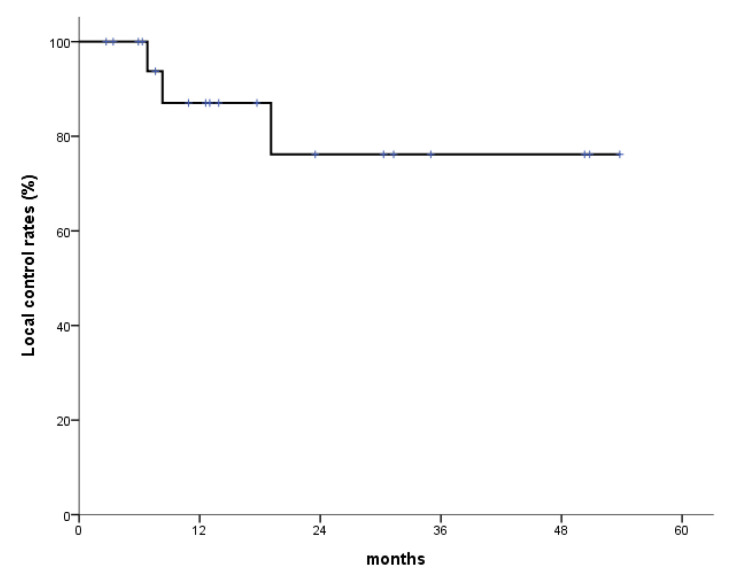
Local control (LC) rates. One-year and 2-year LC rates are 87.1% and 76.2%, respectively.

The median follow-up period in the study was 15.8 months (range: 2.7–53.8 months). During follow-up, 10 of the 20 patients (50.0%) showed a complete response (CR), five (25.0%) showed a partial response (PR), two (10.0%) had stable disease (SD), and three (15.0%) had progressive disease (PD). Overall, the response rate during follow-up was 75%. Fourteen patients were alive with no evidence of disease or with controlled disease until the last follow-up. One-year and 2-year OS rates were 79.4% and 62.4%, respectively, and the corresponding LC rates were 87.1% and 76.2%, respectively (Figure 1 and Figure 2). Eight patients died due to a non-radiation therapy cause, including six who died due to disease progression.

One patient died of a treatment-related grade 5 hemoptysis 6 months after radiotherapy (patient no. 19). No grade 3 or higher complications were observed in any patient except in patient no. 19; therefore, the rate of grade ≥3 complication was 5%. Two patients experienced grade 2 esophageal pain 3 months after the completion of radiotherapy, but they recovered well after the administration of pain medication. Two patients had grade 2 radiation pneumonitis, which occurred 3 and 8 months after the completion of SBRT, respectively. Both patients showed improvement after the administration of oral steroids (Table 2). The remaining patients safely completed SBRT and did not experience any significant RT-related toxicities.

### 3.3. Complete Response

Among the patients with CR, 5 patients were treated with a definitive aim, 4 patients were treated with a salvage aim, and the remaining 1 patient received consolidation SBRT for SCLC. Among the patients with CR, patient no. 20 was a 64-year-old male with severe dyspnea due to underlying emphysema, and surgery was not easy because he was also taking an anticoagulant after percutaneous intervention (PCI) with non-ST-segment-elevation myocardial infarction (NSTEMI). He achieved CR at 6 months after 55 Gy in 10 fractions of SBRT. Patient no. 6, the only SCLC patient in this study, initially had extensive disease (ED) with brain, left subaortic and hilar lymph node metastases. Chemotherapy (etoposide/cisplatin regimen) was initiated first, but treatment was discontinued due to grade 4 neutropenia. Therefore, 30 Gy in 10 fractions of whole brain RT (WBRT) was given to the brain, and SBRT of 55 Gy in 10 fractions was given to the lung lesions. He showed CR at 6 months after the completion of lung SBRT, and CR in brain at 3 months after WBRT. However, 2 years and 10 months after the end of WBRT, a 6 mm sized mass recurred in the left cerebellum, so re-RT of 40 Gy in 10 fractions was performed on the brain lesion, and 6 cycles of second-line irinotecan/cisplatin palliative chemotherapy were additionally administered. Currently, both lung and brain are no evidence of disease (NED) status until 50.3 months of follow-up period after the end of first SBRT. All 10 patients with CR had no toxicity of grade 3 or higher, 5 patients with grade 1 radiation pneumonitis, 2 patients with grade 1 cough, and 2 patients with grade 2 radiation pneumonitis, and all recovered well after treatment.

### 3.4. Partial Response

Among the 5 patients with PR, 3 received SBRT for salvage aim and 2 were definitive aim. Patient no. 13 was a 65-year-old male with biopsy-proven squamous cell of lung cancer, cT2N1M0. He was a 60 pack-year ex-smoker. The 2.7 cm enhancing mass was located in the left upper lobe and interlobar lymph node areas, with endobronchial involvement. Therefore, he was recommended left pneumonectomy, but the patient refused surgery. SBRT of 60 Gy in 10 fractions was performed, and PR was shown at 3 months after the end of SBRT. After the SBRT, the patient underwent 4 cycles of chemotherapy (vinorelbine/cisplatin regimen) and maintained PR status. He had no toxicity after SBRT. Among the patients who received SBRT with salvage aim, patient no. 14 was a stage IV NSCLC patient with initial bone and liver metastases. This patient was a 69-year-old woman who was previously healthy and had never smoked. She initially showed a good response after palliative chemotherapy (pemetrexed) for cT3N3M1c of NSCLC, but she had left lower lobe endobronchial-involving mass causing obstructive pneumonitis and atelectasis. This mass was a conglomerated mass at left hilar/interlobar/subcarinal area. She underwent 45 Gy in 10 fractions of SBRT and PR was achieved. She had no toxicities other than grade 1 radiation pneumonitis at 3 months after treatment. Patient no. 3 also showed PR after receiving salvage SBRT. This patient was a 53-year-old male with no specific medical history other than hypertension (HTN). He initially underwent right upper lobectomy due to right upper lobe single mass, and was observed without additional treatment. Surgical pathology revealed NSCLC, adenocarcinoma, and pT2aN0M0. However, one year post-surgery, recurrence of the abutting mass in the right main bronchus was observed with bone metastasis, indicating rcT2N0M1. Therefore, after 60 Gy in 10 fractions of SBRT, PR was shown at 2.7 months after RT, but acute respiratory distress syndrome (ARDS) due to disease progression occurred, resulting in multiple organ failure and death at 2.73 months after the completion of SBRT. Among all 5 patients with PR, one patient (no. 19) had grade 5 hemoptysis (will be described in detail in the next section). In the remaining patients, one experienced grade 1 radiation pneumonitis and 3 patients did not experience any toxicities.

### 3.5. PD and a Case of Fatal Hemoptysis

**Figure 3:**
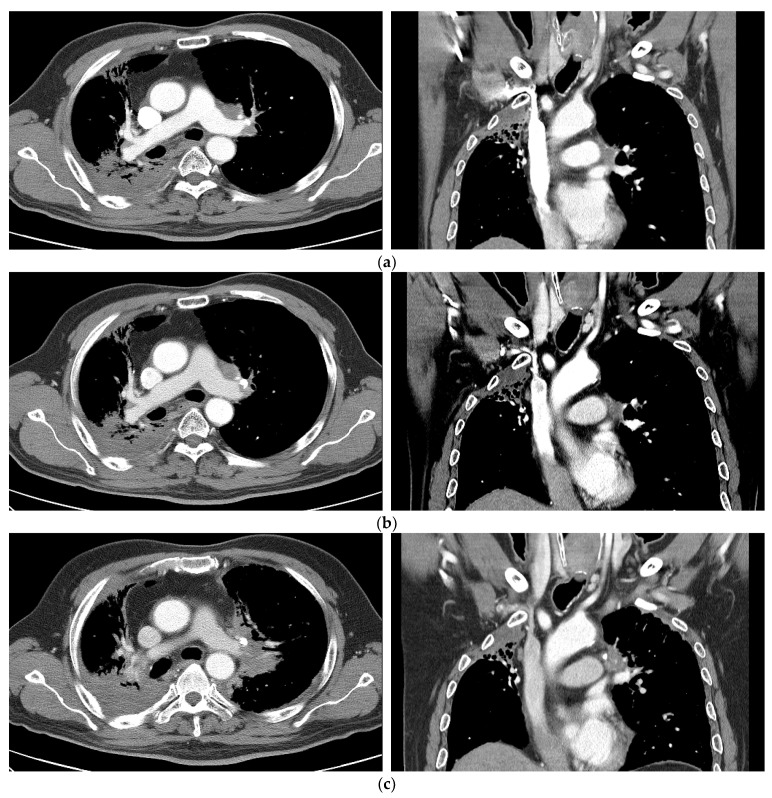
Case 1 (patient no. 7). (**a**) A 62-year-old man, a non-smoker with underlying emphysema, was initially diagnosed with NSCLC of stage cT3N3M0. He underwent definitive CCRT (taxol/cisplatin regimen) of 60 Gy in 30 fractions as the first treatment. Two years and 3 months after CCRT, recurrence at the left prevascular and hilar lymph nodal area occurred. He was recommended to undergo chemotherapy, and underwent re-RT with SBRT at 55 Gy in 10 fractions. (**b**) Follow-up imaging 3 months after SBRT showing SD. (**c**) Occurrence of PD 1 year and 7 months after SBRT. NSCLC, non-small cell lung cancer; SBRT, stereotactic body radiotherapy; SD, stable disease; PD, progressive disease; CCRT, concurrent chemoradiotherapy; RT, radiation therapy.

**Figure 4:**
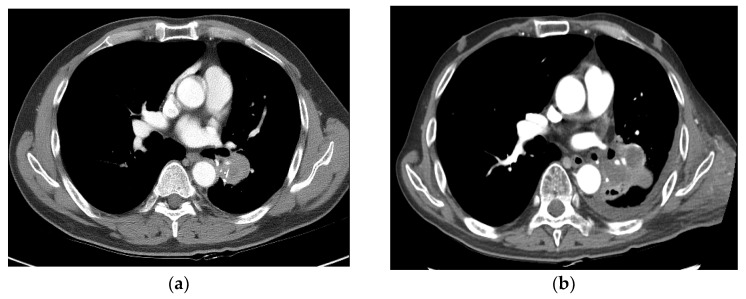
Case 2 (patient no. 15). (**a**) A 58-year-old man was initially diagnosed with NSCLC of stage pT2bN0M0 (stage IIA) after left lower lobectomy. A recurrent tumor at the LLL operation site (left subaortic area) was noted. He underwent SBRT at 50 Gy in 10 fractions. (**b**) Follow-up imaging at 6.8 months showing PD after SBRT. NSCLC, non-small cell lung cancer; SBRT, stereotactic body radiotherapy; PD, progressive disease; LLL, left lower lobe.

Among the 20 patients, three (15.0%) exhibited progressive disease (PD), and all of them underwent salvage treatment. Patient no. 7 was a 62-year-old man, a non-smoker with underlying emphysema, who was initially diagnosed with NSCLC of stage cT3N3M0. He underwent definitive concurrent chemoradiotherapy (CCRT) [taxol/cisplatin regimen] at 60 Gy in 30 fractions as the first treatment; 2 years and 3 months after completion of the first RT, the UC tumor recurred in the left perivascular and hilar areas. Re-RT with SBRT at 55 Gy in 10 fractions was implemented, resulting in SD at 3 months after SBRT. However, PD occurred at 1 year 7 months after SBRT (Figure 3). Palliative chemotherapy (nivolumab) and palliative RT were performed to manage spine and brain metastasis developed 1 year after the completion of SBRT. Despite continued treatment, the patient died 19.1 months after SBRT due to the pneumonia and respiratory acidosis caused by disease progression. Patient no. 11 was a 62-year-old male patient with underlying chronic obstructive pulmonary disease (COPD). He was initially diagnosed with NSCLC of stage cT4N3M0, with a right lower-lobe primary mass, and was treated with definitive CCRT (placlitaxel/cisplatin regimen) at 64.5 Gy in 30 fractions. However, 1 year and 3 months after the first RT, re-RT with SBRT at 55 Gy in 10 fractions was performed due to the recurrence of a UC tumor in the right middle lobe/right lower lobe. However, signs of progressive disease (PD) were evident on follow-up imaging 8.3 months after completion of SBRT. Thereafter, he was treated with atezolizumab; however, because of lymph node metastasis, the treatment approach was changed to gemcitabine/carboplatin chemotherapy. Since then, he has been receiving navelbine treatment until the last follow-up due to left upper lobe recurrences. Patient no. 15 was a 58-year-old man, non-smoker, who was initially diagnosed with NSCLC of stage pT2bN0M0 (stage IIA) after left lower lobectomy. He underwent four cycles of adjuvant chemotherapy (taxol/cisplatin regimen). Ten months after the first surgery, video-assisted thoracic surgery (VATS) wedge resection (WR) was performed due to LUL recurrence. Six months after WR, he was treated with SBRT at 50 Gy in 10 fractions due to UC lung tumor recurrence at the LLL postoperation site (left subaortic area) (Figure 4). At 6.8 months post-completion of SBRT, evidence of progressive disease (PD) was observed in the SBRT treatment area as well as in the lung, bone, pleura, muscle, and brain. Conservative treatment with brigatinib was started. However, the patient died of multiple organ failure due to disease progression at 9.1 months after SBRT. One of the two patients with PD had grade 2 radiation pneumonitis, while the other had grade 1 radiation pneumonitis. No grade 3 or higher toxicity was observed in any patient with PD.

**Figure 5:**
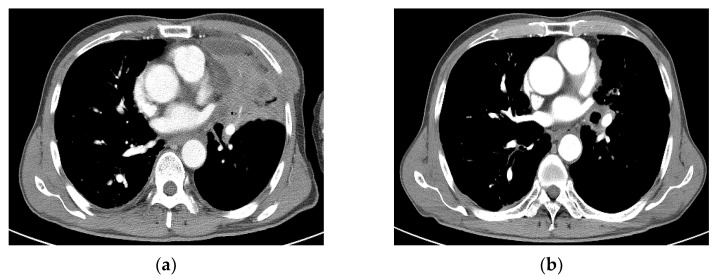

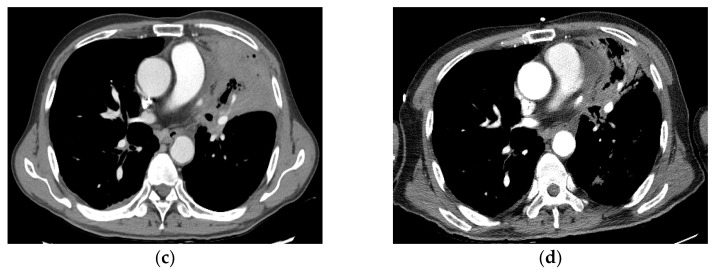
Case 3 (patient no. 19). (**a**) A 68-year-old man with obstructive pneumonitis had a left upper lobe mass obstructing the left upper lobe segmental bronchus. He also had pseudoaneurysm of the pulmonary artery. He was recommended to undergo pneumonectomy. He underwent SBRT at 55 Gy in 10 fractions because of underlying pneumoconiosis and COPD. (**b**) Follow-up imaging at 2 months showing PR after SBRT. (**c**) Chest CT 4 months after SBRT showing tumor necrosis with progression of radiation pneumonitis/tracheobronchitis or combined infection, with diffuse wall thickening of the trachea and left main bronchus, accompanied by pericardial effusion. (**d**) Follow-up imaging 6 months after SBRT showing improved pneumonitis and aggravation of the dilated pulmonary artery inside the consolidation in the left upper lobe. However, massive hemoptysis was observed 6.3 months after SBRT, which resulted in death. CT, computed tomography; SBRT, stereotactic body radiotherapy; PR, partial response; COPD, chronic obstructive pulmonary disease.

One patient died of treatment-related grade 5 hemoptysis 6 months after radiotherapy (patient no. 19) (Figure 5). He was a 68-year-old man who had underlying pneumoconiosis, COPD, and history of old pulmonary tuberculosis 30 years ago. A left upper lobe mass obstructed the left upper lobe segmental bronchus, and the patient was recommended to undergo pneumonectomy. Before SBRT, he also had pseudoaneurysm with dilated pulmonary artery inside the consolidation in the left upper lobe. On bronchoscopic biopsy, the tumor was confirmed to be SqCC. However, the patient had underlying lung disease, making it difficult to perform surgery. He underwent SBRT at 55 Gy in 10 fractions and achieved PR 2 months after SBRT. Massive hemoptysis was observed 6.3 months after SBRT with aggravated pseudoaneurysm. Hemoptysis was estimated to be approximately 2–4 cups, along with a cough, sputum production, and shortness of breath. The patient showed loss of consciousness when admitted to the emergency department. After intubation, bronchial artery embolization was performed twice. However, he expired due to respiratory arrest.

## 4. Discussion

SBRT has shown similar levels of LC and survival outcomes as those observed with surgical resection for localized tumors [2]. SBRT is widely acknowledged as the alternative option for inoperable cases with localized lung cancers. As tumors abutting the main bronchus (i.e., UC tumors) commonly require extensive surgery, including pneumonectomy or sleeve lobectomy, non-invasive and possibly curative SBRT may confer a significant therapeutic benefit. However, SBRT was not recommended for UC tumors previously due to the risk of fatal hemorrhage [5]. Early studies reported a non-negligible occurrence of treatment-related toxicities. Tekatli et al. [14] reported a grade 5 complication rate of 21.2% (14.9% due to hemorrhage) after SBRT for treating UC tumors, and Haseltine et al. [15] also reported four cases of fatal hemorrhage (22.2%) among 18 treated cases. In the study by Tekatli et al. [14], 60% of the tumors were larger than 5 cm, and planning target volume (PTV) D_max_ was greater than 123% in all treatment plans. Haseltine et al. [15] claimed that two of the four cases were due to infectious pneumonia, and two cases might have been related to the use of bevacizumab. Recent studies have reported a much lower rate of fatal toxicities. Recent large-scale studies have reported grade 5 toxicity rates between 2 and 5% [16,17,18]. In the updated meta-analysis by Rim et al. [6], the pooled rate of grade 3 complications was 9% and that of fatal toxicity was 5.7%. Pooled rates of LC and OS were 90.4% and 66.4% at 2 years, respectively, and showed the clinical significance of SBRT for the treatment of UC tumors. Updated 2023 National Comprehensive Cancer Network (NCCN) guidelines stated that centrally located and even UC tumors could be effectively and safely treated with protracted SBRT [3].

In the present study, LC and OS rates at 2 years were 76.2% and 62.4%, respectively. Although the survival outcome was similar to that reported in previous studies, the LC rate was lower than that reported in recent studies (>90% at 2 years) [6,16,17,18]. We hypothesized that the prescribed dose (median: 55 Gy in 10 fractions, 82.5 Gy_10_ in biologically equivalent dose [BED]) was relatively lower than that used in recent studies. Meng et al. [17] reported a 2-year LC rate of 92%, with a prescribed median dose of BED 120 Gy_10_. Chang et al. [18]. reported a 2-year LC rate of 96.6% after prescribing a median dose of BED >100 Gy_10_ in 92% of the included patients. However, dose escalation should be done cautiously because high-dose SBRT, when treating UC tumors, may result in fatal toxicities. Tekatli et al. [14]. prescribed a median dose of BED 132 Gy_10_, with median D_max_ of 138%, resulting in 21.2% of grade 5 toxicities. Although one case of fatal hemoptysis was reported in our study (5%), no other grade 3 complication was reported, and only three patients experienced grade 2 complications. Employing moderate-dose radiation therapy and implementing constraints on excessive dosages (with D_max_ <110% in all protocols) during planning could influence the toxicities observed in the study. Considering that two-thirds (65%) of the patients included in our study received SBRT for salvage purposes aiming to address recurrent disease, it is presumed that this may have influenced toxicity outcomes. Additionally, among these patients, two individuals underwent re-RT following CCRT, which further suggests a potential impact on toxicity.

Unfortunately, we encountered one case of a fatal hemorrhage. The 5% grade 5 complication rate (1 of 20) correlated with that reported in a recent meta-analysis (5.7%) [6]. The patient had pseudoaneurysm of the pulmonary artery before SBRT, which was encased in the tumor. It was temporally resolved after 1 month of SBRT and recurred at 5 months thereafter with cancer progression. Considering the pre-existing presence of a pseudoaneurysm prior to treatment, the occurrence of a hemorrhage presents a nuanced aspect where causality directly related to the treatment or otherwise remains uncertain. Pseudoaneurysm of the pulmonary artery due to lung cancer is rare but can cause fatal hemoptysis [19]. Squamous histology and treatment-induced tumor necrosis are presumed to be the main causes associated with hemoptysis [19,20]. Conventional radiotherapy to treat centrally located lung cancer can yield fatal hemorrhage at a rate of 13–17% [21,22,23]. While various factors such as radiation dose, fractionation scheme, endobronchial involvement, and the use of bevacizumab could contribute to fatal hemorrhagic toxicity, it also suggests that a pseudoaneurysm may also contribute to toxicity. Although hemorrhagic toxicity is the most concerned complication of SBRT for central tumors, any modality of local treatment cannot fully avoid the risk of hemorrhage for cases with pulmonary artery pseudoaneurysm. Therefore, frequent follow-up with imaging studies is necessary, and administration of medications with bleeding tendency should be minimized.

Especially since the advent of new drugs such as durvalumab and pembrolizumab in 2021, there has been a tendency to prioritize chemotherapy/immunotherapy over SBRT for UC, which may carry a bleeding risk. Therefore, there is significance in demonstrating the safety and efficacy of SBRT for UC tumors through our study, and there is a need to evaluate the utility of SBRT in the era of immunotherapeutic agents. Cong et al. [24] and Lodeweges et al. [25] mentioned that 3.9% and 2.8% of patients, respectively, received immunotherapy before undergoing SBRT. In our study, out of 20 patients, seven received immunotherapy after SBRT for adjuvant or palliative purposes, and none received it concurrently. Future research should concentrate on examining the synergistic potential of immunotherapy in conjunction with SBRT, evaluating its influence on clinical outcomes and risk profiles, specifically in patients diagnosed with UC.

Our study had some limitations. Due to the small number of patients, we could not achieve statistically significant results to investigate factors affecting clinical outcomes or toxicities. Nevertheless, there is clinical significance in reporting the outcomes of patients with UC tumors who underwent moderate-dose SBRT, particularly regarding long-term complications such as bleeding risk. Our study, being a retrospective single-center study, indeed has a limitation in lacking a comparator group. Given the scarcity of patients who underwent SBRT for UC tumors, we compare our data with that from other historical studies (Table 3). When compared to other historical studies, our study demonstrated comparable results in terms of LC and OS, and showed lower or similar rates of grade 3 or higher toxicity after moderate-dose SBRT. Another limitation is the clinical heterogeneity of the patients owing to the diversity of cancer types and the aim of the treatment (salvage and primary). At our institution, we could not prescribe treatment primarily at diagnosis but recommended SBRT for patients who were deemed not amenable for, or those who refused, other treatments, including upfront surgery or palliative chemotherapy.

## 5. Conclusions

In conclusion, moderate-intensity SBRT is a feasible and effective curative modality, without excessive toxicity, for treating patients with UC lung tumors. Future studies with a larger sample size should be performed to identify factors affecting clinical outcomes and toxicities, thereby optimizing the patient selection criteria for SBRT.

## Figures and Tables

**Table 1:** Patient and treatment characteristics (*n* = 20).

Patient ID	Age (Years), Sex	FU Period	Treatment Aim	Underlying Pulmonary Disease	Smoking Status	Stage	Location	Pathology	Recommended Treatment	Tumor Volume (cm^3^)	SBRT Dose	Response
1	75, F	50.8	Salvage s/p LULobectomy	no	never	rcT0N2	4R LN	ADC	Chemotherapy	7.6	55 Gy/10 fx	SD
2	57, M	31.3	Primary	Tuberculosis (10 years ago)	current (30PY)	cT3N0M0	RUL bronchus	SqCC	Rt pneumonectomy	22.5	50 Gy/10 fx	CR
3	53, M	2.7	Salvage s/p RULobectomy	ILD, NTM	never	rcT2N0M1	Main bronchus	ADC	Chemotherapy	11	60 Gy/10 fx	PR
4	65, M	53.8	Salvage s/p RULobectomy	no	never	rcT0N2	4R LN	ADC	Chemotherapy	5.7	55 Gy/10 fx	CR
5	68, M	7.6	Primary	Emphysema	ex-smoker (20PY)	cT2N0M0	Lt hilar	LCNEC	Lt pneumonectomy	21.3	50 Gy/10 fx	near CR
6	51, M	50.3	Consolidation	no	ex-smoker (15PY)	ED, cT1aN1M1b	Lt subaortic, Lt hilar	SCC	Chemotherapy	4.0	55 Gy/10 fx	CR
7	62, M	19.1	Salvage s/p CCRT (interval 3.5 years)	Emphysema, PTE	Never	rcT0N2M0	Lt prevascular, Lt hilar	SqCC	Chemotherapy	67.4	55 Gy/10 fx	PD
8	66, M	5.9	Primary	no	ex-smoker (28 PY)	cT2N0M0	Lt main/lobar/segmental bronchi of LUL	SqCC	Lt pneumonectomy	25.6	55 Gy/10 fx	CR
9	66, M	30.3	Salvage	tuberculosis	current (55 PY)	rcT0N2M1	LUL Lt prevascular	SqCC	Chemotherapy	7.1	60 Gy/10 fx	PR
10	48, F	35.0	Salvage	no	never	rcT0N2M1	Rt hilar LN	ADC	Chemotherapy	3.6	60 Gy/10 fx	CR
11	62, M	35.3	Salvage s/p CCRT	COPD	never	rcT2N0M0	RML/RLL central	SqCC	Chemotherapy	29.1	55 Gy/10 fx	PD
12	73, M	13.9	Salvage	no	never	rcT2N2M0	RUL Rt hilar	ADC	Chemotherapy	5.2	55 Gy/10 fx	near CR
13	65, M	12.6	Primary	no	ex-smoker (40 PY)	cT2N1M0	LUL Lt Interlobal LN	SqCC	Lt pneumonectomy	47.2	60 Gy/10 fx	PR
14	69, F	12.5	Salvage	no	never	ycT3N3M1c	LLL bronchus	ADC	Chemotherapy	13.0	45 Gy/10 fx	PR
15	58, M	9.1	Salvage	no	never	rcT4N0M0	LLL	ADC	Chemotherapy	42.2	50 Gy/10 fx	PD
16	57, M	10.9	Salvage s/p LULobectomy	no	never	rcTxN2M0	Lt hilar LN	LCNEC	Chemotherapy	22.4	55 Gy/10 fx	near CR
17	64, M	3.4	Salvage s/p LULobectomy	no	never	rcT2N3M1b	Lt subaortic LN	ADC	Chemotherapy	87.4	55 Gy/10 fx	SD
18	77, M	13.0	Primary	no	ex-smoker (30 PY)	cT2bN0M0	LUL with LLL invasion	NA	Lt pneumonectomy	28.9	60 Gy/10 fx	near CR
19	68, M	6.3	Primary	Pneumoconiosis, COPD, old Tb (40 years ago)	ex-smoker (30 PY)	cT2N0M0	LUL	SqCC	Lt pneumonectomy	29.0	55 Gy/10 fx	PR
20	64, M	17.7	Primary	COPD	ex-smoker (30 PY)	cT2N0M0	LUL	SqCC	Lt pneumonectomy	21.9	55 Gy/10 fx	near CR

FU = follow up; M = male; F = female; SBRT = stereotactic body radiation therapy; PY = pack-year; LN = lymph node; SqCC = squamous cell cancer; ADC = adenocarcinoma; LCNEC = large cell neuroendocrine carcinoma; SCC = small cell carcinoma; NA = not assessed; Lt = left; Rt = right; LUL = left upper lobe; RUL = right upper lobe; CR = complete response; SD = stable disease; PR = partial response; fx= fraction; NTM = nontuberculous mycobacterial lung disease; COPD = chronic obstructive pulmonary disease; ILD = interstitial lung disease; PTE = pulmonary thromboembolism.

**Table 2:** Patient and treatment risk factors and complications (*n* = 20).

Patient ID	Endobronchial Involvement	Anticoagulant Use	Bevacizumab Use	D_max_ (%)	RT Complications (≥Grade 2)
1	No	No	No	106.0	Late grade 2 esophageal pain (3 months after SBRT)
2	Yes	No	No	107.4	None
3	No	Yes	No	104.9	None
4	No	No	No	106.3	None
5	Yes	Yes	No	108.1	None
6	No	No	No	102.1	Grade 2 radiation pneumonitis (3 months after SBRT)
7	No	No	No	107	None
8	Yes	Yes	No	106.1	None
9	No	No	No	108.2	None
10	No	No	No	106.2	None
11	No	No	No	106.8	Grade 2 radiation pneumonitis (8 months after SBRT)
12	No	No	No	105.2	None
13	Yes	No	No	105.3	None
14	Yes	No	No	106.3	None
15	No	Yes	No	106.2	None
16	No	No	No	105.2	None
17	No	No	No	105.8	None
18	No	No	No	109.5	None
19	Yes	No	No	105.6	Grade 5 massive hemoptysis (6 months after SBRT)
20	Yes	No	No	104.7	None

RT = radiotherapy; SBRT = stereotactic body radiation therapy.

**Table 3:** Published reports of the SBRT for UC lung tumors.

Study	No. of Pts (Lesions)	FU Period (Median, Months)	Tumor Location	Radiation Regimen (Gy/fx)	2-Year LC (%)	2-Year OS (%)	Toxicity (%, ≥Gr3)	Characteristics
Tekatli et al. (2016) [14]	47	29.3	UC	60/12	100	20.1	38	38% were stageIIIA, 17% recurrent
Haseltine et al. (2016) [15]	108 18	22.7	Central and UC	45/5	-	-	12	Primary lung tumor (81%) and recurrent (19%)
Chang et al. (2018) [18]	61 46	16.7	Central vs. UC	30–49/5 or ≥50/5	3.4 4.3 (LFR)	57.7 50.4	3.5 8.7	Metastatic lung cancer
Cong et al. (2019) [24]	51	17.0	UC	35/5	54.5 (1yr)	-	9.8	Advanced UC-NSCLC
Nguyen et al. (2019) [26]	(69) (14)	19.7	Central and UC	40–60/3–8	85	72	14.2 (≥Gr2) 14	Central, UC and paramediastinal tumors
Lenglet et al. (2019) [16]	(60) (77)	36	Central and UC	40–60/3–8	81	Median 57 37	7.3	Primary or recurrent lung cancer
Park et al. (2019) [8]	8	7.6	UC	50–60/10	100 (1-year)	87.5 (1-year)	0	Primary and recurrent
Lodeweges et al. (2021) [25]	72	19	UC	60/12	85	52	21	Early stage inoperable lung cancer
Loi (2021) [27]	109	17	UC	50/5 or 45/6 or 48–60/8 or 50–70/10	78	55	6	UC Oligometastasis (≤3) 66% NSCLC 13% Colorectal cancer
Breen et al. (2021) [28]	110	30	UC	50/5 or 60/8 or 48/4	16 (LP)	57	Acute 5 Late 8	Ablative, curative intent RT for UC NSCLC
Current study (2024)	20	15.8	UC	45–60/10	76.2	62.4	5	Primary (45%) and recurrent (55%) NSCLC

SBRT = stereotactic body radiation therapy; UC = ultra-central; No. = number; FU = follow up; LC = local control; OS = overall survival; PFS = progression-free survival; LFR = local failure rate; LP = local progression NSCLC = non-small cell lung cancer.

## Data Availability

The datasets used and/or analyzed during the current study are available from the corresponding author on reasonable request.
